# Recent Progress in Intelligent Wearable Sensors for Health Monitoring and Wound Healing Based on Biofluids

**DOI:** 10.3389/fbioe.2021.765987

**Published:** 2021-11-01

**Authors:** Siyang Cheng, Zhen Gu, Liping Zhou, Mingda Hao, Heng An, Kaiyu Song, Xiaochao Wu, Kexin Zhang, Zeya Zhao, Yaozhong Dong, Yongqiang Wen

**Affiliations:** ^1^ Beijing Key Laboratory for Bioengineering and Sensing Technology, Daxing Research Institute, School of Chemistry and Biological Engineering, University of Science and Technology Beijing, Beijing, China; ^2^ School of Material Science and Engineering, Zhengzhou University, Zhengzhou, China; ^3^ Yantai Yuhuangding Hospital, Yantai, China

**Keywords:** intelligent, wearable, health monitoring, biofluids, targeted analytes, wound healing

## Abstract

The intelligent wearable sensors promote the transformation of the health care from a traditional hospital-centered model to a personal portable device-centered model. There is an urgent need of real-time, multi-functional, and personalized monitoring of various biochemical target substances and signals based on the intelligent wearable sensors for health monitoring, especially wound healing. Under this background, this review article first reviews the outstanding progress in the development of intelligent, wearable sensors designed for continuous, real-time analysis, and monitoring of sweat, blood, interstitial fluid, tears, wound fluid, etc. Second, this paper reports the advanced status of intelligent wound monitoring sensors designed for wound diagnosis and treatment. The paper highlights some smart sensors to monitor target analytes in various wounds. Finally, this paper makes conservative recommendations regarding future development of intelligent wearable sensors.

## Introduction

The development and design of wearable intelligent sensors have led to excellent potential applications in the fields of public health monitoring and health care. Although this field is in its infancy, the foundational research in the interdisciplinary area of wearable sensing is well established. The preparation of novel devices tends to be tightly integrated with the development of various emerging technologies, including biocompatible materials (He S. et al., 2020); flexible electronics (Zhang et al., 2019); optical and electrochemical sensors (Gao et al., 2016a; Gao et al., 2016b; Nyein et al., 2016); microfluidics (Kim et al., 2020); near-field communication (NFC) (Kim et al., 2015); painless microneedles (Guo et al., 2019; Li et al., 2019); big data; and cloud computing (Yang et al., 2016). In addition, intelligent, wearable sensors are often associated with the human body in the form of tattoos; patches; and gloves or dressings. Live sensing, data recording, and computing have been performed using external devices and portable systems (Wang C. et al., 2018; Ershad et al., 2020; Hanna et al., 2020). Compared with the traditional medical health monitoring that relies on a typical medical infirmary, the intelligent biochemical sensors have attracted ever-increasing attentions for their flexibility, rapidity, biocompatibility, high specificity, and low cost (Kim et al., 2019; Bocchetta et al., 2020; Koralli and Mouzakis, 2021). More importantly, this technology creates two-way feedback between doctors and patients to develop more personalized and scientific health care programs (Hughes, 2008). It can monitor specific information-carrying molecules and signals that are related to human physiology and disease pathology and output data about target analytes to external terminal equipment continuously and in real time, thus aiding in prediction and diagnosis (Dervisevic et al., 2020). Efficient acquisition of target analytes is achieved by collecting biofluids that are naturally secreted by or are part of the human body, including sweat, tears, skin interstitial fluid (IFS), blood, and wound fluids (Kassal et al., 2017; Li and Wen, 2020). Below, we discuss some biofluids for providing physiological and pathological information.

Sweat is the fluid secreted by sweat glands and contains a number of biomolecular and biochemical signaling analytes. Sweat is also a representative source of analytes in the field of intelligent wearable sensors. The analytes include ionic electrolytes (Na^+^, K^+^) (Gao et al., 2016a), metabolites (glucose, lactate) (Valdes-Ramirez et al., 2014; Koh et al., 2016), and heavy metal species (copper, iron, zinc) (Sekine et al., 2018).

Blood distributes throughout the body and typically requires being invasively collected prior to analysis and monitoring in wearable sensor devices. Some advanced blood sensing devices have been developed recently for continuous detection of health care (He S. et al., 2020; Hanna et al., 2020).

Interstitial Fluid (ISF) is another source of biomarkers such as protein and glucose in serum and plasma (Koschinsky et al., 2003; Tran et al., 2018). ISF is often collected and analyzed using microneedle patch (Ventrelli et al., 2015).

Tears’ composition is not as complex as that of blood, due to the presence of the blood-tear barrier. Tears often contain analytes similar to other biofluids, including glucose, Na^+^, and K^+^ (Tseng et al., 2018). Contact lens-based portable sensors enable tear collection efforts, but sophisticated sensor design concepts are often required (Kim et al., 2017c; Liu et al., 2021).

Wound fluid is derived from various types of skin, mucosal surface or organ tissue injuries and consists of a highly inhomogeneous mixture whose physicochemical markers are assumed to reflect the clinical status of the wound healing (Löffler et al., 2013). The wound biomarkers include biochemical molecules [C-reactive protein, potential hydrogen (pH), glucose, uric acid, etc.] (Pasche et al., 2008; Kassal et al., 2015; Jankowska et al., 2017; Pan et al., 2019), biochemical signals (such as wound temperature, pressure, and redox state) (Farooqui and Shamim, 2016; Sun et al., 2018), and pathogens (such as *Pseudomonas aeruginosa*) (Brunauer et al., 2021). Wound monitoring is a key challenge for the next generation of smart dressing development.

Wounds are inevitably caused by internal factors (such as chronic diseases) (Siddiqui and Bernstein, 2010) or external factors (such as mechanical and thermal) (Demidova-Rice et al., 2012; Zahedi et al., 2010). It is usually painful for patients to face wounds with stress. It is urgent to develop effective wound management systems and strategies to promote complex wounds healing. The wound healing stages include hemostasis, inflammation, proliferation, and remodeling (Aderibigbe and Buyana, 2018; Gonzalez et al., 2016). Table 1 shows the repair characteristics of the various wound healing stages. Any abnormal or incomplete recovery stage can lead to wound healing delays (Nunan et al., 2014). In clinics, wound-related information indicators are often monitored using visual examination and laboratory analysis of exudate swabs, but the efficiency of this type of wound care is typically low and advanced preventative nursing methods are needed.

**TABLE 1:** The four stages of wound healing and their characteristics.

Hemostatic stage	Interconnection between blood vessels slows blood flow to the injured tissue and minimizes bleeding caused by the wound (Furie and Furie, 2008; Rodrigues et al., 2019). Platelets can activate and release transforming growth factor (TGF- α, TGF- β), platelet derived growth factor, and other important active factors, which aid subsequent healing stages (Pool, 1977)
Inflammatory stage	Neutrophils are the first cells to arrive at the wound. Their phagocytic ability can effectively remove damaged matrix material, dead cells, and other foreign bodies. Monocytes are recruited subsequently. They differentiate into macrophages and dendritic cells in various tissues. The differentiated cells induce the release of signal factors, which can initiate apoptosis or infection protection, and then effectively remove tissue debris and coagulum (FrykbergRobert and Banks, 2015; Kratofil et al., 2017)
Proliferation stage	The release of Interleukin-1 (IL-1) and tumor necrosis factor-α (TNF-α) can stimulate fibroblast secretion (Kiwanuka et al., 2012). Keratinocytes migrate to the wound surface after replacing dead cells. Granulation tissue formation not only can promote the maturation of keratinocytes, but also can stimulate the release of signal transforming factors, transform fibroblasts into muscle fibroblasts, and complete cell epithelialization (Gonzalez et al., 2016)
Remodeling stage	Type III collagen can be transformed into more stable type-I collagen gradually under the action of matrix metalloproteinases (MMPs). Fibroblasts also migrate to the wound site, deposit new type-I collagen, and eventually complete epidermal tissue replacement or scar tissue repair (Gurtner et al., 2008; Guo and Dipietro, 2010)

The intelligent wearable sensors provide unprecedented data and convenience to support the rational management of patient wounds. These intelligent dressing sensors provide real-time information about wound characteristics for doctors and users. In addition, the collected wound biomarkers data can be transferred to external devices by wireless real-time communication to perform *in-situ*, multi-parameter, and real-time monitoring and healing of a wound (Foster and Sethares, 2014). Therefore, patients no longer need to bear medical expenses related to wound prevention, care, surgery, and long-term hospitalization.

In this review, we first introduce the latest progress in the application of intelligent wearable senors in the fields of health monitoring *via* analysis of various biofluids (sweat, blood, interstitial fluid, tears, wound fluid, etc.). Second, for various types of wound models (infected wounds, chronic wounds, and acute wounds), we review the monitoring of various biomarkers (biochemical molecules, biochemical signals, etc.) in wound fluids. Finally, we make concluding comments and conservatively analyze prospects for the future and the limitations of ideal modern wearable devices.

## Intelligent Wearable Sensors

**FIGURE 1:**
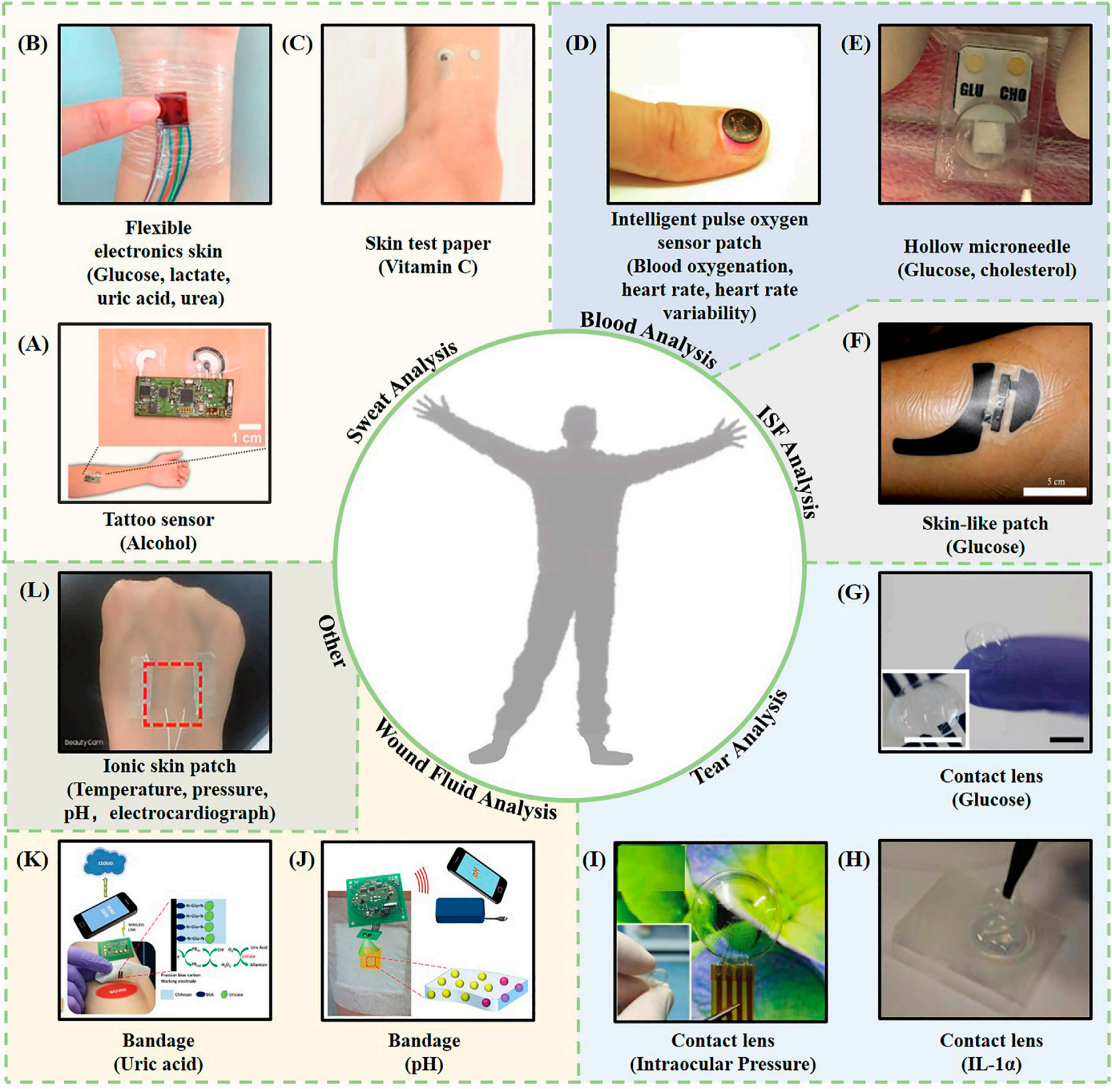
Representative examples of intelligent wearable sensors based on biofluids. **(A)** A wearable tattoo sensor for alcohol detection in sweat (Kim et al., 2016). **(B)** A flexible electronic skin based on a piezoelectric biosensor matrix for monitoring of lactic acid, glucose, uric acid, and urea (Han et al., 2017). **(C)** A skin test paper used for vitamin C monitoring (Sempionatto et al., 2020). **(D)** An intelligent pulse oxygen sensor patch that outputs information such as the blood oxygen, heart rate, and heart rate variability (Kim et al., 2017b). **(E)** A one-touch-activated blood multidiagnostic system (OBMS) for monitoring blood glucose and cholesterol (Li et al., 2015). **(F)** A skin-like patch for noninvasive blood glucose monitoring (Chen et al., 2017). **(G)** A contact lens sensing device for monitoring blood sugar (Kim et al., 2017c). **(H)** A thermotherapy recessive lens for monitoring IL-1α (Mak et al., 2015). **(I)** A contact lens sensor capable of continuous intraocular pressure monitoring (Liu et al., 2021). **(J)** A smart bandage for optical monitoring of pH (Kassal et al., 2017). **(K)** An intelligent bandage for monitoring the uric acid index in a wound (Kassal et al., 2015). **(L)** An ionic bioelectronic skin patch for monitoring of temperature, pressure, pH, and electrocardiogram data (Shi and Wu, 2021).

In modern medical nursing, the medical characteristics of preventive, predictive, personalized, and participatory medicine (the 4P medical model) are the focus of advanced wearable devices (Lin, 2019). Intelligent wearable medical sensors can accurately sense patient pathophysiological information and monitor patient physiological statuses in real time. Specifically, wearable sensors detect target analytes in various biological body fluids (sweat, interstitial fluid, blood, tears, wound fluid, etc.) *in vitro* and *in vivo* for health monitoring (Sturgeon et al., 2008; Jia et al., 2012; Gromov et al., 2013; Broza et al., 2015; Heikenfeld et al., 2019; Shrivastava et al., 2020). These biomarkers include physiological metabolites (Han et al., 2017; Tur-Garcia et al., 2017; Mishra et al., 2018; Choi et al., 2019), small molecules (Munje et al., 2017; Parlak et al., 2018; Li et al., 2021), biochemical factors (Mak et al., 2015), and environmental signals (Pal et al., 2020; Liu et al., 2021; Shi and Wu, 2021). This section systematically summarizes the latest applications of intelligent wearable sensors for monitoring biomarkers in various biofluids (Figure 1) as summarized in Table 2.

**TABLE 2:** Representative new intelligent wearable sensor platforms based on biofluids.

Biological fluid sampled		Material or platform	Targeted analyte	Detection limit	Biosensing format	Ref
Biofluid	Sweat	Sweatband	Zn, Cd, Pb, Cu, Hg	NR	Electrochemical-stripping voltammetry	Gao et al. (2016a)
		Microfluidic patch	Lactate, glucose	50 µM (Glucose)	Electrochemical-amperometry	Martin et al. (2017)
		Polyamide film	Glucose, cortisol	0.1 mg/dl (Glucose)	Electrochemical-amperometry and impedance	Munje et al. (2017)
		Polycarbonate membrane	Lactate	0.2 mM	Electrochemical-amperometry	Tur-Garcia et al. (2017)
		Flexible electronics skin	Glucose, lactate, uric acid, urea	NR	Piezoelectric system	Han et al. (2017)
		Microfluidic patch	Lactate, pH, glucose, and chloride	NR	Colorimetry	Koh et al. (2016)
		Temporary tattoo	Alcohol	NR	Amperometry	Kim et al. (2016)
		Stretchable patch	Glucose, pH	1.3 μM (Glucose)	Amperometry	Oh et al. (2018)
		Microfluidic patch	Na+	NR	Potentiometry	Nyein et al. (2018)
		PANi-Nafion-OPH/PVA hydrogel	Diisopropyl fluorophosphates	NR	Potentiometry	Mishra et al. (2018)
		Glucose colorimetric assay kit	Glucose	NR	Colorimetry	Choi et al. (2019)
		In_2_O_3_- Au/chitosan-SWCNT/GOx	Glucose	10 nM	Field-effect transistor	Liu et al. (2018)
		3D-printed e-ring bridges	Glucose	1.2 μM	Electrochemical-amperometry	Katseli et al. (2021)
		Skin test paper	Vitamin C	NR	Amperometry	Sempionatto et al. (2020)
	Blood
		Intelligent pulse oxygen sensing ring	Blood oxygenation, pulse rate	NR	Optoelectronic system	Lochner et al. (2014)
		PEGDA	Lactate	1 μM	Cyclic voltammetry	Caliò et al. (2016)
		Intelligent pulse oxygen sensing patch	Blood oxygenation heart rate, heart rate variability	NR	Electrochemical-amperometry	Kim et al. (2017b)
		Hollow microneedle	Glucose and cholesterol	NR	Colorimetry	Li et al. (2015)
	Interstitial fluid	Embroidered bandage	Glucose, lactate	NR	Electrochemical-amperometry	Liu and Lillehoj (2016)
		Epidermis sensing gloves	Glucose	NR	Electromagnetic system	Hanna et al. (2020)
		PtNps/PANi/MEA/GOx	Glucose	260 µM	Cyclic voltammetry	Gao et al. (2019a)
		PtNps/PANi/MEA/UOx	Uric acid	4 µM		
		PtNps/PANi/MEA/ChOx	Cholesterol	440 µM		
		Skin-like patch	Glucose	NR	Amperometry	Chen et al. (2017)
		Microneedle patch	Methyl paraoxon	4 µM	Amperometry	Mishra et al. (2017)
		Microneedle patch	Glucose	NR	Amperometry	Li et al. (2021)
		Microneedle patch	Glucose	0.66 mM	Electrochemical-amperometry	Dervisevic et al. (2021)
	Tears	AuMNA- P(GMA-co-VFc)	Urea	2.8 µM	Cyclic voltammetry	Senel et al. (2019)
		Contact lens	Glucose	0.4 mM	Field-effect transistor	Kim et al. (2017c)
		Contact lens	Glucose	12.57 mM	Field-effect transistor	Park et al. (2018a)
		Contact lens	Interleukin-1α (IL-1α)	1.43 pg/ml	Amperometry	Mak et al. (2015)
		Contact lens	Intraocular Pressure	3.166 mV mm Hg^−1^ (on the porcine eye)	Amperometry	Liu et al. (2021)
	Wound fluid	Bandage	Uric acid	NR	Amperometry	Kassal et al. (2015)
		Bandage	pH	6.5–8.5	Optical	Kassal et al. (2017)
		Bandage	Tyrosinase	NR	Amperometry	Ciui et al. (2018)
		Wound dressing	pH	6.0–9.0	Colorimetry	Pan et al. (2019)
		Wound dressing	Reduction state	NR	Colorimetry	He et al. (2020a)
		Epidermal electronics system	Temperature, thermal conductivity	NR	Amperometry	Hattori et al. (2014)
		Intelligent conductive hydrogel	Large deformation movement of human body	NR	Amperometry	Zhao et al. (2019)
Other	Epidermis				Amperometry	Ershad et al. (2020)
		Temporary tattoo	Moisture, heart rate	NR	Electrochemical-amperometry and impedance	Shi and Wu (2021)
		Ionic skin patch	Temperature, pressure, pH,electrocardiograph	NR	Ultrasound wall-tracking technique,	
		Flexible monitoring patch	Blood pressure	NR	Electrochemical-amperometry	Wang et al. (2018a)

### Basic Composition and Design Principles for Intelligent Wearable Sensors

Intelligent wearable sensors often exist in biological and chemical-based sensing modes. A typical biochemical sensor includes three essential functional elements: 1) Substrate for integrating complete sensors; 2) A “receptor probe” element that can selectively identify target analytes; 3) A signal output element that converts an event recognized by the receptor into a readable signal (electrochemical mode, optical mode et al.) (Bandodkar and Wang, 2014; Kim et al., 2019). Suitable substrate materials in intelligent wearable sensors often utilized polyethylene terephthalate (PET), polyethylene (PE), and inkjet temporary tattoo paper (Bandodkar et al., 2015; Gao et al., 2016a; Wang J. et al., 2018). However, their poor air permeability and tensile properties limit the miniaturization of the sensors. Biodegradable flexible materials such as cellulose, wool, polyacrylonitrile (PAN) and poly (styrene-butadiene-styrene) (PSBS) fiber are usually more attractive as substrates (Bandodkar et al., 2015; Kim D. et al., 2017; Wu et al., 2017; Marriam et al., 2018).

Selecting and fixing an appropriate receptor probe on the substrate are the most critical step for intelligent wearable sensors, and the design principles are as follows: 1) The substrate used for fixing receptor probe cannot interact with the target analyte; 2) To stabilize the receptor probe on the substrate, it is usually necessary to modify the substrate and probe with appropriate functional groups or introduce intermediate connecting media; 3) The fixation must be reliable, repeatable, and capable of ensuring the biochemical activity of the receptor probe to achieve efficient detection of the target analyte.

Signal output elements can quantify target analytes and provide convertible or intuitive signals, including electrochemical and optical sensing. For example, the target analyte will undergo an oxidation-reduction reaction with the electrode when the electrochemical sensor is working, thereby generating a small current (Curto et al., 2012). Specific enzyme amperometric sensors (lactate oxidase, urate oxidase, and urate oxidase) are often used to monitor lactic acid, uric acid, and lactic acid. The sensor can measure the potential between the working electrode and the reference electrode in the electrochemical cell. The method of conductance measurement usually adopts the mode of field-effect transistor (FET) (Bandodkar et al., 2019). Its sensing principle can be summarized as follows: the channel current between the source and drain of the sensor varies with the charge density which is sensitive to the target material on the surface of the transistor (Sang et al., 2016). If the material used in the channel is graphene, it is called a graphene field-effect transistor (GFET) (Prattis et al., 2021). For example, a peptide nucleic acid (PNA) based GFET quantitatively detected RNA target with the graphene (Tian et al., 2020). The PNA probe with an amine bond frame provided specific binding efficiency to DNA and RNA targets, thus reducing the detection time (Mat Zaid et al., 2017; Movilli et al., 2018). Under the global COVID-19 epidemic background, a team has developed a GFET sensor with an anti-virus spike protein antibody for rapid detection of severe acute respiratory syndrome coronavirus (SARS-CoV-2). The lowest detection limit is 1 fg/ml (Seo et al., 2020).

Electronic sensing devices often require continuous power supply. Optical sensors based colorimetric or fluorescence analysis modes consume almost no energy. Compared with amperometric sensing devices, colorimetric and fluorescent sensors also have the advantages of simple structure, low cost, and portable design without power supply operation (Zhu et al., 2021). For example, fluorescence biosensors consist of excitation light sources (lasers), fluorophore molecules, and photodetectors for fluorescence intensity and spectrum recording (Chen et al., 2017). Among them, fluorophore molecules include small molecules, proteins, nucleic acids, etc., (Jensen, 2012). In addition to the above-mentioned sensing technology, microfluidics technology become prominent in wearable intelligent sensor platforms for reducing samples and solvents (Li et al., 2020; Mejía-Salazar et al., 2020).

### Sweat

**FIGURE 2:**
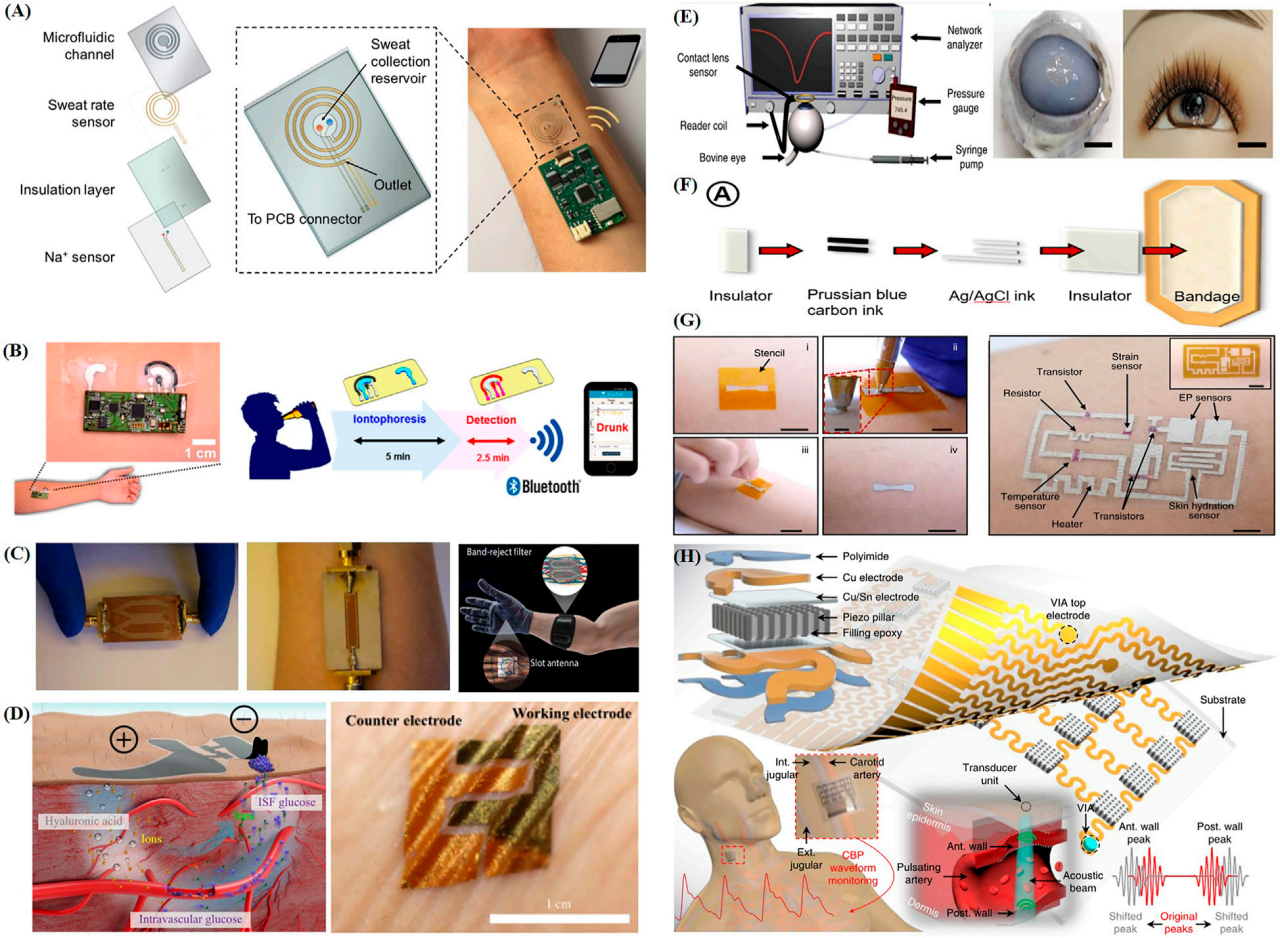
Recent progress of representative intelligent wearable sensors designed for health monitoring. **(A)** Schematic diagram of the structure and principle of a wearable sweat patch (Nyein et al., 2018). **(B)** Schematic illustration of the application of a skin tattoo-based wearable alcohol sensor patch to the skin and its wireless sensing principle (Kim et al., 2016). **(C)** Optical image of a wrist and back of hand based on an electromagnetic wave-sensing wearable device that imitates the vascular anatomy (Hanna et al., 2020). **(D)** Schematic diagram of a wearable skin biosensor system for non-invasive electrochemical monitoring of glucose in ISF (Chen et al., 2017). **(E)** Schematic of a contact lens sensing device used to monitor blood glucose and intraocular pressure and its appearance when fitted (Kim et al., 2017c). **(F)** Schematic diagram of screen printing bandage sensor preparation (Kassal et al., 2015). **(G)** Schematic illustration of the DoS circuit preparation process and structure (Ershad et al., 2020). **(H)** Schematic diagram of the structure and operation principle of a wearable flexible monitoring patch that uses ultrasound (Wang C. et al., 2018).

Sweat is relatively easy to obtain and rich in biochemical information (Such as biomolecules, inorganic salts, metal elements, etc.) for non-invasive monitoring of the wearer’s physiological status. Sempionatto’s team developed a flexible vitamin C tattoo patch sensor to monitor the time distribution of vitamin C levels in sweat by attaching ascorbate oxidase (AAOx) to a flexible, printable tattoo electrode patch (Sempionatto et al., 2020). Excessive cortisol molecules can lead to the development of diabetes, so it is necessary to measure cortisol indicators in the body through a portable method (Jones and Gwenin, 2021). Parlak et al. (2018) reported a wearable senor with a molecularly selective organic electrochemical transistor layer can collect 10–50 μl of sweat at a time and evaluate cortisol levels accurately. Electrolyte imbalance can cause abnormal health conditions of the human body and change the composition of sweat. Nyein et al. (2018) proposed a flexible microfluidic sweat sensor patch (Figure 2A), in which a commercially printed circuit board (PCB) and a sweat rate sensor based on electrochemical and electrical impedance were integrated into the microfluidic channel. The device can be used to monitor Na+ concentration and sweat rate signals simultaneously.

Continuous operation of intelligent sensors usually requires strict energy supply quality (Park S. et al., 2018). Recently, investigators demonstrated that a wearable flexible microarray sensor provided multi-channel real-time detection of trace heavy metals (Zn, Cd, Pb, Cu, and Hg) in sweat (Gao et al., 2016b). Ordinary batteries often suffer from insufficient availability and battery life (Zamarayeva et al., 2017; Zeng et al., 2014). Obtaining energy directly from bioenergy including human body heat and biomoleculars are potential sources of power for the sustainable development of intellegent sensors in the future (Barros, 2013; Dienel and Hertz, 2001). Yu et al. (2020) proposed a biofuel-driven sensing electronic skin that consisted of a multimode sensor and a high-efficiency lactic acid biofuel cell. The lactic acid present in the sweat was catalyzed by the lactate oxidase in the anode of the electronic skin to generate pyruvate, and the cathode reduces the oxygen in the environment to water. The stable current generated in the process could realize the self-powering of the sensor. The electronic skin could also selectively monitor physiological information such as urea, pH, and glucose, as well as body temperature and sweat characteristics in real-time. Unlike using human endogenous biofuels (glucose or lactate secreted by the human body) for power generation, another work reports a flexible portable epidermal biofuel cell based on human exogenous substances (Sun et al., 2021a). The device consists of two parts, one is a sweat transport microfluidic module for sampling, storage and excretion, the other part is a non-invasive real-time biological organism *in situ* generations of flexible ethanol/oxygen biofuel cell module. The epidermal biofuel cell uses exogenous substances (such as ethanol) instead of endogenous substances as a new generation of promising energy-saving products, which can be used for sweat collection and sweat power generation on the skin of drinkers at the same time. To prevent and monitor unsafe drinking behavior, Kim et al. (2016) developed a wearable alcohol sensor patch based on a skin tattoo (Figure 2B). The patch consisted of an iontophoretic-biosensing temporary tattoo system and flexible electronics. It was coated with the drug pilocarpine. Drugs can be released through the skin in *via* iontophoresis, which induced the epidermis to produce sweat. Alcohol oxidase and Prussian blue electrode transducer can be used to monitor alcohol molecules in sweat.

Glucose in sweat is reported to be associated with blood glucose metabolism (Talary et al., 2007). Studies have reported an attachable, expandable electrochemical sensor that could continuously monitor blood glucose concentrations and pH changes caused by eating, exercise, or disease for long periods (Oh et al., 2018). Katseli et al. (2021) developed a wearable electrochemical ring (e-ring) to self-test the blood glucose level in human sweat upon connecting the smartphone to a micro-potentiostat by 3D printing technology. Xiao et al. (2019) developed a wearable colorimetric sensor based on a microfluidic chip, in which five microfluidic channels guided sweat into a detection microcavity. Each channel had a check valve to ensure that the reagent could not flow back from the microcavity (Xiao et al., 2019). The colorimetric response of the enzymatic oxidation of o-dianisidine supplied the detection of 0.10–0.50 mM glucose with a detection limit of 0.03 mM in sweat. The field-effect transistor (FET), a new type of sensing device, has attracted substantial attention in the field of sensing because of its low manufacturing cost, industrial production, and high sensitivity (Syu et al., 2018). The principle of a FET biosensor can be summarized as follows: the channel current between the source and drain of the sensor varies with the charge density which is sensitive to the targeted substances on the specific surface of the transistor (Sang et al., 2016). Liu et al. (2018) developed a highly sensitive In_2_O_3_ nanobelt based on FET biosensing. It integrated FET biosensors with an on-chip gold side gate and offered good electrical properties on highly flexible substrates (Liu et al., 2018). Further research has shown that the device can detect glucose concentrations in the range of 10 nM–1 mM, and its sensitivity is sufficient to cover the range of abnormal glucose content in human biological fluids caused by diabetes. Although sweat monitoring has many advantages in the field of sensing, problems such as the inability to obtain sweat immediately in winter and easy contamination of sweat are still constraints and challenges for the development of clinical diagnosis.

Although great progress has been made in sweat analysis with intelligent wearable sweat sensors, there are also some key challenges. 1) The perspiration rate is related to the season, so the actual efficacy of the sensor is unstable throughout the year; 2) Sweat is exposed to the outside world when it is secreted, and it is easy to be contaminated, thus affecting the results of sweat analysis. 3) Somatic species lack volume control over sweat evaporation and collection. Solving these problems requires breakthroughs in sweat collection and transportation, such as the development of new materials as well as novel overall encapsulation strategies (Heikenfeld, 2016).

### Blood

Blood is the liquid in the circulatory systems of humans and higher animals. It transports oxygen and nutrients (glucose, amino acids, and acids), removes wastes (carbon dioxide, uric acid, lactic acid), and provides immune and information functions. Blood tests contribute to detecting internal health at the level of cytology, and providing reasonable health care suggestions (Hallek et al., 2008; Peters et al., 2004). A variety of devices have been developed to monitor target analytes in blood (Lee et al., 2009; Lee et al., 2011; Zheng et al., 2015). Lochner et al. reported a flexible substrate-compatible sensor composed entirely of organic optoelectronic devices for measuring the human pulse and arterial oxygen partial pressure with errors of 1 and 2%, respectively (Lochner et al., 2014). Li et al. (2015) reported an intelligent one-touch-activated blood multidiagnostic system (OBMS) for detecting the glucose and cholesterol. Within 3 min, one press of the finger started the diagnostic, including human blood collection, red blood cell separation, serum transportation and detection. Liu and Lillehoj (2016) reported a dual electrochemical sensor prepared by embroidery technology with simultaneous detection of glucose and lactate at high-sensitivity. In recent years, wearable devices tend to be miniaturized, battery-free, lightweight, and noninvasive. Kim et al. (2017b) reported a millimeter-scale and battery-free pulse oximeter mounted on the fingernail for capturing quantitative information such as the blood oxygen level, heart rate, and heart rate variability. Another study proposed a blood glucose measurement sensor system based on electromagnetic waves (EM) and integrated it with gloves for noninvasive detection of glucose in the blood (Figure 2C) (Hanna et al., 2020). The working microwave frequency band of the sensor is designed between 500 MHz and 3 GHz. The microwave energy in this frequency range reaches the vessel across the epidermis and muscle tissue layer with high sensitivity, ensuring that the sensor monitors glucose in a wide frequency range. The results of a controlled experiment in diabetic mice and healthy humans showed that the physical characteristic information fed back by the sensor system has a highly linear relationship with the measured blood glucose level (>0.9). The intelligent non-invasive blood glucose monitoring system can avoid the discomfort caused by acupuncture, optimize the patient’s medical experience, and also bring great chances to medical diagnosis and health management. Besides sensor devices driven by biofuel cells used in sweat, Sun et al. (2021b) reported a biofuel cell-driven medical nanodevice used in serum, which includes two parts: a vitamin C sensor chip (iezCard) for self-powered energy and an output chip for signal processing. The iezCard in the device integrates a special Kimwipes (A common laboratory paper) microfluidic channel, which can achieve efficient transmission of the serum to be tested. In addition, the Kimwipes microchannel has a filtering effect on proteins, and the device can directly detect vitamin C from the serum. The device uses a drop of serum to realize the immediate detection of scurvy caused by the lack of vitamin C. In another study, to improve human immunodeficiency virus (HIV) testing, a side-flow detection platform based on microfluidic fuel cells was developed, which includes a biological anode that can be used to oxidize glucose in the blood and a biological cathode that can be used to reduce the transport of oxygen in the air (Dector et al., 2017). The biological anode is composed of methylene blue electropolymerization paper deposited with tetrabutylammonium bromide deionized water, glutaraldehyde, Nafion, and glucose oxidase. The biological cathode is composed of Pt/C on methylene blue electropolymerized paper. This work proves that the fuel cell integrated in HIV side flow detection has considerable real-time testing potential in the energy field. Blood is still the most critical biological fluid in personal health monitoring. To sum up, blood is still an essential biological fluid in personal health monitoring. Its screening can not only provide a basis for disease diagnosis, curative effect diagnosis, and post-medical prediction of the blood system but also provide an essential reference for the diagnosis and treatment of diseases that cause secondary changes in blood composition. It seems excessive to rely on intrusive sensing methods to monitor glucose and vitamin C, as sensors for sweat analysis are also reported to be used for related molecular monitoring. Therefore, blood sensor detection should monitor target analytes not found in other biological fluids—for example, CA125 markers of ovarian cancer and cardiac troponin markers suspected of an acute coronary syndrome (Saorin et al., 2020). Advanced blood sensors will rely on more serious power supply technical problems. Commercial coin batteries are the most widely used but have disadvantages in weight, volume, and rigid mechanical properties. To overcome this problem, the biofuel cell outlined above is considered an effective way to generate electricity *in situ*.

### ISF

ISF is a combination of serum and cellular material, produced *via* transcapillary filtration of blood and cleared by lymphatic vessels (Wiig and Swartz, 2012). ISF includes small molecular metabolites such as salt, protein, glucose, and ethanol, much like blood (Fogh-Andersen et al., 1995; Tran et al., 2018). Therefore, ISF is an ideal blood substitute for medical health monitoring, as it offers convenient collection, sustainable monitoring, non-coagulation, and good applicability to the field of sensing (Kastellorizios and Burgess, 2015).

Chen et al. (2017) developed a skin-like biosensor system for non-invasive blood glucose monitoring. The system consisted of an ultra-thin, skin-like biosensor and electrochemical twin channels (ETCs) that functioned as a paper battery. ETCs increased the ISF osmotic pressure *via* iontophoresis and drove glucose in blood vessels to be transported to the skin surface, thus achieving sensitive and accurate glucose monitoring (Figure 2D). In agricultural environments, farmers are often exposed to organophosphorus (OP). Because organophosphorus pesticides are easily absorbed by the skin and highly toxic, there is an urgent need for fast, sensitive, and reliable OP sensing tools. Mishra et al. developed an invisible microneedle sensing system based on organophosphorus hydrolase (OPH) (Mishra et al., 2017). A carbon paste electrode transducer was used to wrap the hollow microneedles and couple the biocatalytic OPH with the sensor. The enzyme reaction products on epidermal sample were sensitized and detected using fast square-wave voltammetry in the presence of OP. Studies have shown that the OPH microneedle sensing system can directly, quickly, and selectively detect methyl paraoxon products in the 20–180 μM range in ISF. Dervisevic et al. reported a type of high-density silicon microneedle array patch for *in-situ* monitoring of blood in ISF (Dervisevic et al., 2021). Another study proposed an integrated wearable closed-loop system based on mesoporous microneedle iontophoresis with a diabetes treatment system (Li et al., 2021). In addition to the minimally invasive extraction of ISF in the form of microneedles mentioned above, ISF can also be extracted to the skin’s surface by reverse iontophoresis or ultrasonic introduction (Leboulanger et al., 2004; Yu et al., 2012). However, similar to sweat sensing, contamination in the process of collecting ISF will affect the accuracy of sensing, so it is necessary to develop more innovative and refined extraction methods.

### Tears

Tears are transparent water forms secreted by lacrimal glands and conjunctival goblet cells. They contain a variety of chemical components, such as water, proteins, electrolytes, sugars, and organic acids (Butovich, 2008; Pankratov et al., 2016). The dynamic balance of the various components within tears ensures the health of individuals. Changes in tear composition can also be used to predict some disease information (Sempionatto et al., 2019). For example, breast cancer patients express a complement protein which is different from non-patients (Evans et al., 2001). Continuous monitoring of tear glucose concentrations can be used in the adjuvant treatment of diabetes (Sen and Sarin, 1980; von Thun und Hohenstein-Blaul et al., 2013). Elevated intraocular pressure is the risk factor for glaucoma and lead to blindness in severe cases (Leonardi et al., 2004; Mansouri et al., 2012; Chen et al., 2013). These indicate analysis of the eye microenvironment including specific analytes in tears is crucial means for long-term, non-invasive monitoring of human health.

Herpes simplex virus serotype-1 (HSV-1) is a major infectious disease that causes blindness in people all over the world (Carr et al., 2001; Streilein et al., 1997). From infection to virus activation and then to disease, patients exhibit signs of corneal scar formation, thinning, neovascularization, etc., accompanied by inflammatory reactions (Hamrah et al., 2010; Liesegang, 2001). Therefore, it is vital to identify and monitor enough biomarkers to predict the pathological state before the virus is activated in the patient. Mak et al. (2015) adopted a facile layer-by-layer (LBL) surface engineering technique to develop a hyperthermic recessive lens with a bifunctional hybrid surface that could be used to interfere with the activity of HSV-1. The contact lenses offered excellent surface wettability and optical transparency, and were non-toxic to human corneal epithelial cells (HCECs). Furthermore, the device promoted high analytical sensitivity to interleukin-1α, and the detection limit was 1.43 pg ml^−1^. Kim et al. developed a contact lens with a built-in wireless smart sensor (Figure 2E) (Kim et al., 2017c). The contact lenses include highly stretchable, transparent graphene sheets, and metal nanowires, which endowed the glasses with high transparency (>91%) and elongation (∼25%), thus ensuring good eye comfort and visual patency. The intraocular pressure (IOP) was measured by a non-conductive dielectric layer. As the intraocular pressure increasesd the radius of corneal curvature became larger. IOP sensors embedded in contact lenses can detect this and send information to a wireless antenna. In addition, tear glucose monitoring was performed using a highly sensitive FET biosensor. Liu et al. (2021) designed and fabricated an ultra-sensitive contact lens sensor to continuously monitor IOP. The contact lens was formed by compounding a uniform graphene film on a flexible polyimide substrate *via* face-to-face water transfer technology. Its average sensitivities on silicone eyes and pig eyes were 1.0164 mV mmHg^−1^ and 3.166 mV mmHg^−1^, respectively. Another study developed an intelligent flexible contact lens by integrating glucose sensor, wireless energy transmission circuit and wireless sensor signal display (Park J. et al., 2018). The sensor contained graphene channels on which glucose oxidase (GOD) was immobilized. When the channels were soaked with tears, glucose molecules and their reduction products could be oxidized by GOD and oxygen molecules in turn to generate main carriers such as protons and electrons. The density of main carrier was positively correlated with glucose concentration which can be detected by establishing the function of relative change of sensor resistance and glucose concentration. The sensing contact lens can detect tears with glucose concentration greater than 0.9 mM, and the minimum detection concentration is 12.57 μM. Contact lens sensor is a suitable tear monitoring platform. It does not cause any irritation to the eyes but can continuously contact with tears and does not need to provide a liquid collection device based on an ISF analysis sensor (Pankratov et al., 2016). Besides glucose monitoring, non-invasive monitoring with MMP-9 in tears as a nonspecific inflammation analyte may improve the diagnosis of eye inflammation (Chotikavanich et al., 2009; Wei et al., 2013). The levels of tear cytokines Th1 and Th17 are usually associated with dry eye disease (Fujishima et al., 2016). The changes in the content of these potential tear target analytes are consistent with those in the blood, it needs to be further verified whether they follow the tear-blood concentration correlation.

### Wound Fluid

Wound management requires optimization by monitoring wound indicators and information-containing molecules in wound fluids. Wearable wound dressings and bandages for real-time monitoring contribute to detecting the wound healing state and evaluating potential follow-up treatments. Changes in uric acid levels are related to the degree of damage to the leg venous ulcer wound and oxidative stress (Fernandez et al., 2012). Kassal’s team prepared uric acid sensor with Prussian blue-carbon electrode on soft dressings by screen printing process, and then combined with a customized wearable potentiostat to develop a smart bandage with wireless capability (Kassal et al., 2015) (Figure 2F). The team also developed an intelligent bandage could optically monitor pH changes at the wound with high precision (Kassal et al., 2017). Pan et al. (2019) developed a simple color-changing fiber sensing material by adding curcumin, a functional biocompatibility indicator, to monitor the pH of a wound in real time. Another study used polyvinyl alcohol (PVA) foams and sodium carboxymethyl cellulose (CMC) nanofibrous membranes as composite substrates filled with stearyl trimethyl ammonium chloride and methylene ammonium bromide to prepare a multilayer net wound dressing (He M. et al., 2020). CMC had certain hemostatic properties because of its irregular reticular structure, while PVA foam had excellent adsorption properties and can repair the wound exudate. Under 650 nm laser irradiation, the killing of bacteria could be achieved by activating the photodynamic reaction of MB and thus generating bactericidal reactive oxygen species. The detailed discussions are in the next section “*Detection and Treatment During Wound Healing*”.

Intelligent wearable sensor devices can provide real-time information about the state of wound lesions. The wound healing cycle is often long, and the sensors based on wound fluids usually cannot work until the wounds are healed and are easily contaminated. Therefore, it requires a high degree of consistency in the sensor’s detecting performance during the healing process. In addition, the development of the sensor with self-cleaning performance is an exciting research direction, which can reduce the number of sensor replacements, production costs and patient compliance.

### Other

In addition to the intelligent bandages and dressings introduced above, wearable sensing devices embedded directly to the surface of the epidermis are another potential future epidermal and wound sensing solution.

In 2014, Hattori et al. (2014) proposed a skin-like epidermal electronic system that could be laminated gently onto the wound and provided accurate real-time monitoring of wound healing in a clinical environment. Several groups of miniature metal resistors in this system could measure the wound surface temperature with multi-mode and high precision. A soft film was covered with miniature metal wires and precision skin temperature measurement and curve determination were achieved using high-end infrared cameras. In addition, the system could record the thermal conductivity of the focus tissue after disinfection. Inspired by the natural structure and function of the skin, Zhao et al. (2019) developed a novel antibacterial conductive hydrogel (PDA@AgNPs/CPHs) made *via* supramolecular assembly of polydopamine-modified silver nanoparticles (PDA@AgNPs), polyaniline, and polyvinyl alcohol. PDA@AgNPs/CPHs not only offer adjustable mechanical and electrochemical properties, good self-healing ability, and repeatable adhesion, but also can monitor large-scale human movements in real time. Further research revealed that the hydrogel could promote wound healing of a diabetic foot *via* promotion of angiogenesis when attached to the mouse model of an ulcerated wound. It accelerated collagen deposition, inhibited bacterial growth, and controlled wound infection. In another study, Ershadetal et al. (2020) proposed a new type of biomedical circuit: a super-conformal drawn-on-skin (DoS) electronic device. It could be used to treat skin wounds of arbitrary shape and to track and monitor physiological signals such as muscle signals, the heart rate, and the skin moisture content (Figure 2G). DoS electronic products are directly “written” on human skin using liquid functional ink to form wearable electronic devices that are super-conformal, expandable, and unaffected by movement. In addition, DoS devices offer stable performance during perspiration, capture electrophysiological signals reliably for long periods, adhere well to skin, and are immune to motion artifacts during sensing. After using the DoS electrode to draw circuits on the backs of depilated mice and perform electrical stimulation, it was found that the degree of wound healing was higher in electrically treated mice than that in untreated mice, thus confirming that skin pulse electrical stimulation driven by skin electronics can promote wound healing. Hydrogel-based multi-functional products have gradually become materials of interest for simulation of human skin perception and provision of protection functions. However, there are few cases of hydrogels with conductive sensing functions that work with interfacial interactions between the environment and hydrogel materials (Ge et al., 2020). For this reason, Lei et al. (2021) proposed a new generation of hydrogel ion skin materials with biomimetic ion channels. These materials could achieve signal transmission between biological and abiotic interfaces and are expected to extend simple skin sensory diagnosis to effective treatment in clinical applications (Lei et al., 2021). A more advanced “intelligently adhered” polyelectrolyte hydrogel (QAAH)-ionized skin based on quaternized chitosan (QCS) was reported in another study. It could be used for medical monitoring of multiple physiological signals (temperature, pressure, pH, and ECG) (Shi and Wu, 2021). The thermal response behavior of QAAH was enabled *via in-situ* polymerization of acrylic acid (AA) monomer in QCS aqueous solution. The pH response behavior is related to the protonation effect of the amino group in QCS. The excellent conductivity, adhesion, and formability are the result of reversible ion association and hydrogen bond physical crosslinking of QAAH. This type of green material, which offers multi-signal resolution and adjustable mechanical and visual effects, has substantial value in clinical medical auxiliary device and intelligent wound management applications. Wang C. et al. (2018) introduced a type of ultrasonic-based wearable flexible monitoring patch that can be in close contact with the skin. It is used for non-invasive, continuous, accurate monitoring of vascular signals in many parts of the human body (Figure 2H). The entire device is assembled layer-by-layer using polyimide, copper electrode, copper/tin electrode, piezoelectric column, and epoxy resin layers. When installed in the human neck, the device can monitor the central blood pressure by capturing the vascular diameters of the carotid artery, internal jugular (int. jugular) vein, and external jugular (ext. jugular) vein. In addition, the device can use a highly directional ultrasound beam to locate the dynamic anterior (ant.) and posterior (post.) walls of blood vessels, and display the corresponding shifting echo radiofrequency signals reflected. This is an example of a new type of conformal telescopic ultrasound equipment that can be used to record a series of key central blood vessel features and is safe and reliable.

There are considerable innovative achievements related to new, intelligent wearable sensors in the field of public health monitoring and medical care. In the future, researchers should focus on the development of multi-functional sensors for all aspects of human physiological information detection and human motion signal tracking. These will help to improve the current medical service and health care system.

## Detection and Treatment During Wound Healing

**FIGURE 3:**
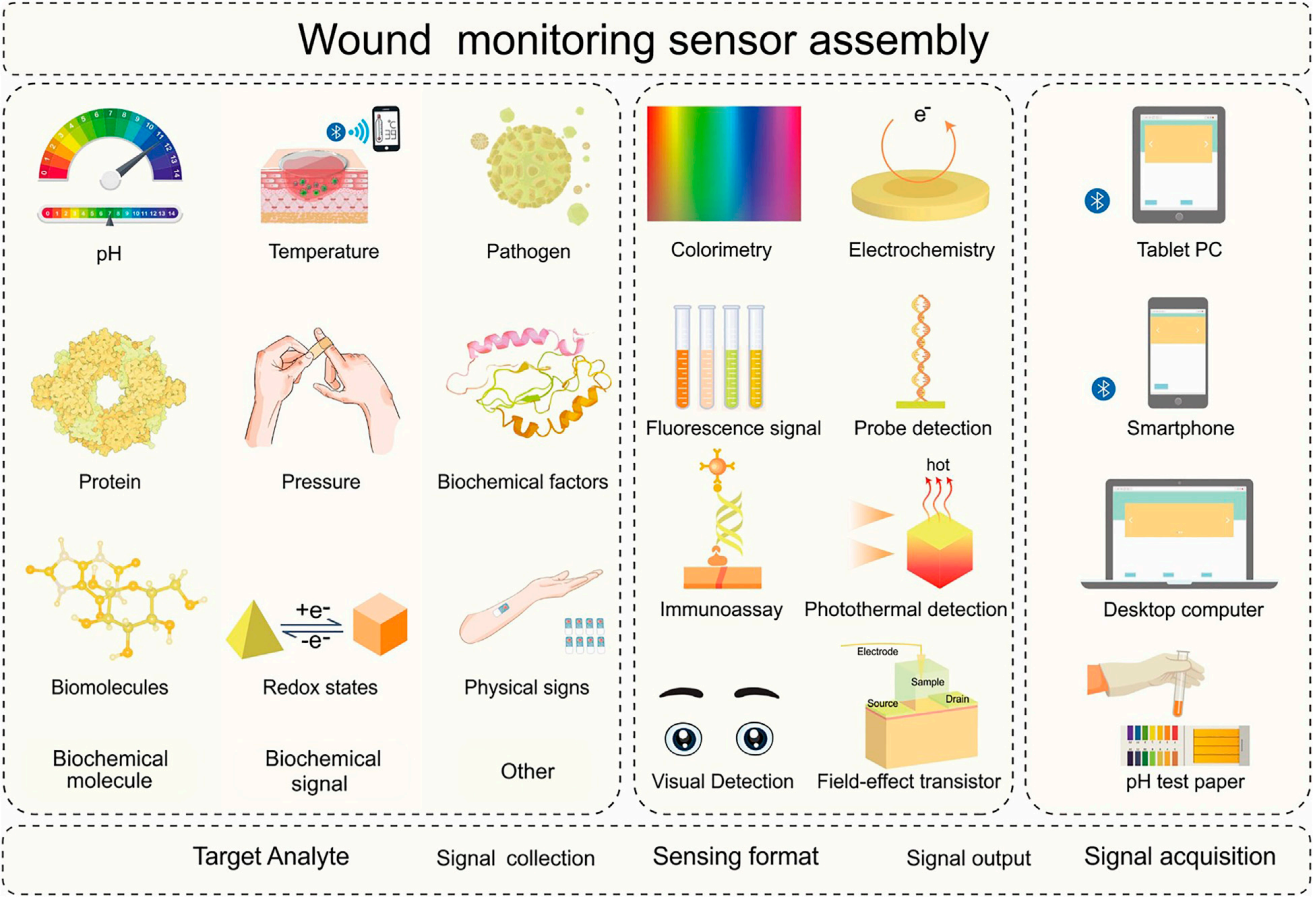
Schematic diagram of the sensing process and integration of the wound monitoring sensor. The target analyte identification element in the wound monitoring sensor can selectively collect various target analytes in different biofluids. The signal processing elements can collect signals using various biochemical sensing formats. The signal acquisition element can process and output analytical results intelligently. The target analytes may include biochemical molecules (potential hydrogen, protein, biomolecules), biochemical signals (temperature, pressure, redox status), and other parameters (pathogens, biochemical factors, physical signs). Sensing formats include colorimetry, fluorescence signal, immunoassay, visual detection, electrochemistry, probe detection, photothermal detection, and Field-effect transistor. Signal acquisition components include pH test strips, computers, tablets, and mobile phones.

The rate of chronic complex trauma diagnoses increases every year worldwide. Health care institutions and hospitals must invest large amounts of resources in the diagnosis and management of wounds. At the same time, wound refractory symptoms caused by traumatic infection, spontaneous ulcers, and other chronic diseases are becoming increasingly common. Patients must bear costly medical expenses related to wound prevention, care, surgery, and long-term hospitalization (FrykbergRobert and Banks, 2015; Sen et al., 2009). The skin is the human organ with the largest surface area. It serves to regulate the body temperature and repairs itself automatically (Vig et al., 2017). Exudate flows out when the skin is injured. During different stages of wound healing, the exudate may contain various biomolecules (such as potential hydrogen, glucose, uric acid, and glutathione), biochemical factors (such as tumor necrosis factor-α and interleukin-6), and pathogens (such as *Pseudomonas aeruginosa*). Their concentrations and properties provide key information about the state of the wound. In addition, wound healing is accompanied by changes in several biological signals (redox state, pressure, temperature), which can be used to predict wound development and trauma-related changes. A typical wound monitoring sensor is composed of target analyte identification, signal processing, and signal acquisition elements (Figure 3). Wearable devices based on various sensing modes are widely used to monitor the wound environment and exudate markers (McLister et al., 2014; Steinberg et al., 2016), including screen-printed electrode potential sensing, cyclic voltammetry sensing, linear fast voltammetry sensing, amperometric sensing, and colorimetric sensing (Guinovart et al., 2014; Steinberg et al., 2015; Kafi et al., 2019) (Table 3).

**TABLE 3:** Representative new intelligent sensor platforms for wound healing.

	Target analyte		Material or platform	Wound type	Detection limit	Biosensing format	References
Wound monitoring sensor	Biochemical molecule	pH, C-reactive protein (CRP)	Integrated portable system	Acute wound	6–8 (pH)	Optical signal	Pasche et al. (2008)
					1 µg/ml (CRP)		
		pH, Glucose	Fluorescence sensing system	Chronic wound	6.0–7.7 (pH)	Fluorescence signal	Jankowska et al. (2017)
					2.5 Mm (Glucose)		
		pH	Wound dressing	Acute or chronic wound	NR	Colorimetry	Mirani et al. (2017)
		pH	Wound dressing	Acute or chronic wound	2–11	Colorimetry	Cui et al. (2020)
		Uric acid	Wound dressing	Simulated wound fluid	NR	Amperometry	Liu and Lillehoj (2017)
		Uric acid, Ph	Bandage	Pressure ulcers	0.2 mM (Uric acid)	Amperometry	Pal et al. (2018)
					5.5–8.5 (pH)		
		Uric acid	Bandage	Chronic wound	NR	Amperometry	RoyChoudhury et al. (2018)
		Glucose, cell proliferation rate	Thin flexible patch Spatially sensitive	Diabetic wound	10 mM (Glucose)	Cyclic voltammetry, linear swift voltammetry	Kafi et al. (2019)
	Biochemical signal	Glutathione	Hydrogel system	Chronic wound	NR	Visual detection	Gao et al. (2019b)
		Redox states	Wound dressing	Acute wound	NR	Probe detection	Sun et al. (2018)
		Pressure	Bandage	Chronic wound	0.1 kPa	Fluorescence signal	Leal-Junior et al. (2021)
		Pressure	Bandage	Pressure ulcers	5 mmHg	Amperometry	Farooqui and Shamim, (2016)
		Temperature	Electronic skins	Infected wound	30–70°C	Amperometry	Gong et al. (2019)
		Temperature	Flexible wound healing system	Infected wound	39–39.5°C	Amperometry	Lou et al. (2020)
		Temperature	Wound Dressing	Infected wound	25–45°C	Amperometry	Pang et al. (2020)
		Temperature	Wound Dressing	Infected wound	25–45°C	Amperometry	Xu et al. (2021)
		Temperature	Flexible integrated sensing platform	Infected wound	33–41°C	Amperometry	Zhang et al. (2021)
		P. aeruginosa	Microfluidic patch	Infected wound	2.1×10^5^ CFU/ml	Flow immunoassay	Brunauer et al. (2021)
	Other	Tumor necrosis factor–α, interleukin-6 (IL-6), IL-8, transforming growth factor–β1	Flexible multi- Venous channel immune ulcer patch	Leg	NR	Electrochemical-amperometry	Gao et al. (2021)
		*S. aureus*	Paper-based biosensor		7 CFU/ml	Colorimetric	Suaifan et al. (2017)

### Biochemical Molecules

**FIGURE 4:**
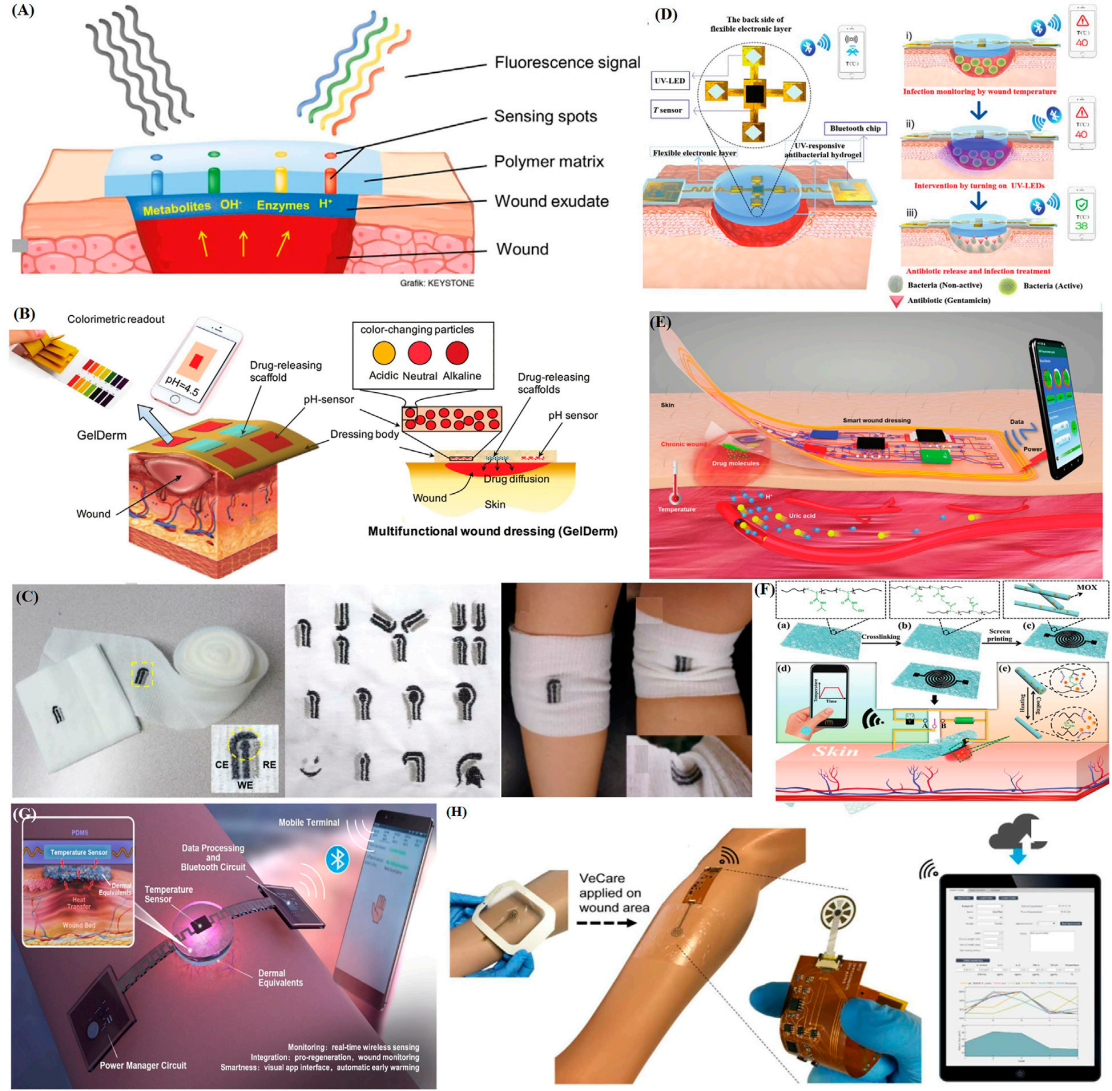
Recent applications of representative intelligent wearable sensors for wound healing. **(A)** Schematic diagram of a fluorescence sensing system with glucose concentration and pH detection (Jankowska et al., 2017). **(B)** Schematic diagram of an intelligent hydrogel dressing (GelDerm) with pH sensitive and drug eluting components for treatment of epidermal wounds (Mirani et al., 2017). **(C)** An electrochemical sensor fabricated on gauze, a wound dressing produced *via* embroidery, and examples of their application to an elbow (Liu and Lillehoj, 2017). **(D)** Intelligent electronic temperature sensing dressing composition and principle schematic diagram (Pang et al., 2020). **(E)** Schematic diagram of a battery-free wireless wound dressing for wound infection monitoring and electronically controlled on-demand wound-site drug delivery (Xu et al., 2021). **(F)** Flow chart showing the preparation of a flexible, breathable skin-based electronic device with temperature sensing capabilities and temperature-based on-demand drug release (Gong et al., 2019). **(G)** Schematic diagram of the structure and sensing principle of a flexible wound healing system (FWHS) (Lou et al., 2020). **(H)** Schematic diagram of a flexible microfluidic multi-immunosensor platform for chronic wound monitoring; a built-in flexible printed circuit board (FPCB) is connected to a wireless portable analyzer and external mobile applications that can be used for patient files, medical records, data records, and data analysis (Gao et al., 2021).

The levels of various biochemical molecules [C-reactive protein, potential hydrogen (pH), glucose, uric acid, etc.] are dynamic balances in the normal skin. However, the wounds break the healthy balance of various biochemical molecules in skin environment. Specific wound types induce responding changes in the levels of biochemical molecules, and these parameters can provide reliable information for evaluating wound healing. The proteins in the wound exudate are closely related with the symptoms. For example, acute-phase proteins such as C-reactive protein (CRP) indicate the presence of infection when the local concentration increases (James et al., 2000). To facilitate the monitoring of biochemical molecules in wounds, Voirin and colleagues developed responsive hydrogels with functional surfaces to monitor pH changes and CRP concentrations, respectively (Pasche et al., 2008). The pH-responsive hydrogel systerm can continuously monitor the pH of serum with the adjustable pH measurement range. In addition, the hydrogel was marked with the optically sensitive CRP receptor. Specific adsorption of CRP leaded to changes in the interfacial refractive index detected by a spectrometer in real-time wound state. An increase in CRP indicated a serious infection and a decrease indicated the end of infection. Jankowska et al. (2017) developed a fluorescent sensor for simultaneous detection of pH and glucose concentrations. It can be used to distinguish the common and chronic wounds during their early stages (Figure 4A). The sensitivity of pH dye for a chronic wound environment reached the range of 6–8. The metabolic enzyme system sensing can identify low glucose concentrations in the exudate of an artificial wound. To optimize the treatment of wound sensors in chronic and complex wounds, Mirani et al. (2017) proposed a smart hydrogel dressing (GelDerm) with a colorimetric pH sensor and drug-eluting stent. It can perform continuous local release of antibiotics without imposing adverse side effects on other organs based on highly accurate inspection of bacterial infection and visual pH detection (Figure 4B). Cui et al. (2020) developed an intelligent wound dressing of alginate fibers with enhanced antibacterial properties and a visual monitoring of wound healing by continuous pH range detection from 2 to 11. Pal et al. (2018) suggested a simple, low-cost, non-invasive wound detection strategy, and prepared omniphobic paper-based smart bandages (OPSBs) by fixing a reusable wearable potentiostat between the adhesive layer and the absorption pad of a commercial bandage to detect pH and uric acid levels simultaneously and communicate the wound status to users or medical staff *via* wireless reports. Liu and Lillehoj (2017) utilized embroidery technology to integrate an electrochemical sensor into a flexible cotton gauze for excellent wound evaluation by continuously detecting uric acid (Figure 4C). Roychoudhury et al. (2018) compounded an enzyme potentiometric biosensor onto the soft cloth of a medical bandage *via* screen printing for sensitive real-time detection of uric acid in wounds as small as 0.5 μl. In addition, the sensing bandage contained a data processing microcontroller for information transmission.

### Biochemical Signals

In addition to biochemical molecules for wounds monitoring, biochemical signals (wound temperature, pressure, and redox state) are also significant for wounds detection and treatment. Sun et al. (2018) prepared a sensor dressing to detect redox state changes during wound healing. First, they successfully constructed a redox-sensitive surface-enhanced Raman scattering (SERS) probe by modifying redox-sensitive anthraquinone molecules on gold nano-shells (GNSSs). Then, the SERS probe was attached to the surface of a chitosan film. Finally, the temporal and spatial evolutions of the wound healing redox state were measured *via in-situ* and non-invasive collection of SERS spectroscopy. The study found that it may be necessary for the redox potential to be minimized during wound healing. Normal wound healing is affected by both the internal pressure of the wound environment and the external pressure exerted by the bandage. The Leal-Junior team proposed a smart bandage based on a highly flexible polymer fiber to evaluate the pressure and pH of the wound area simultaneously (Leal-Junior et al., 2021). The intelligent bandage includes a pH sensitive fiber made from rhodamine B dye-doped polydimethylsiloxane (PDMS) and traditional medical gauze. The low Young’s modulus of the PDMS fiber ensures the high sensitivity of the pressure sensor. The bandage can measure pressures as low as 0.1 kPa and exhibits a highly linear correlation over the 0–0.3 kPa range. Farooqui et al. developed an intelligent bandage using inkjet printing technology to continuously monitor irregular bleeding, pH, and external pressure of the wound (Farooqui and Shamim, 2016). The intelligent bandage assembly includes a disposable tape on which the reusable sensors were printed. The capacitive sensor can detect irregular bleeding-driven changes in the dielectric constant between the electrodes on the two sides of the bandage. Changes in the wound pressure lead to changes in the distance between the electrodes. In addition, the conductivity of the resistive sensor carbon electrode varied with the pH.

Bacterial infection of wounds is an increasingly serious public health problem and imposes large medical and economic burdens. If bacterial reproduction and transfer can be detected and stopped at the early stage, further deterioration of the lesion can be prevented (Ivnitski et al., 1999). Hao et al. (2021) proposed a vancomycin-doped Prussian blue nanoparticle (PB-VANNP) platform that could perform high-sensitivity bacterial detection and avoid secondary pollution by killing bacteria efficiently. The multi-functional nano-platform has the advantages of high sensitivity, low cost, and simple detection. Mostafalu et al. (2018) prepared a smart dressing consisted of two parts: a disposable patch and a reusable pH and temperature sensors with a thermally responsive drug-release bead and a microcontroller. The dressing could indicate the wound state in real time, process sensor data and manage individualized drug release. A recent innovative study demonstrated a drug release dressing controlled by a single exogenous stimulus. The dressing had a double-layer structure in which the upper layer was a flexible electronic device with a temperature sensor and an ultraviolet light-emitting diode encapsulated in polydimethylsiloxane. The lower layer was an ultraviolet (UV)-responsive antibacterial hydrogel (Pang et al., 2020). The wound temperature was monitored continuously using a temperature sensor and transmitted to a foreign terminal device (for example, a smartphone) *via* Bluetooth. When the wound temperature remained above a preset threshold (for example, 40°C) for a period of time, the infected wound was diagnosed and the integrated UV-LED activated to achieve *in-situ* antibiotic release in order to inhibit wound infection. This eventually lowered the wound temperature (Figure 4D **)**. This study combined advanced biomaterials with flexible sensors to provide a new dynamic intervention-based therapy strategy. Xu et al. (2021) used flexible electronic processing technology to construct a wireless intelligent dressing to perform *in-situ*, multi-parameter, and real-time monitoring of a wound and control antibiotic release electronically (Figure 4E). The intelligent dressing can detect changes in physiological parameters such as the pH, uric acid content and temperature of the wound to determine the degree of infection and provide sufficient information for doctors to adjust the treatment plan accurately. The dressing included NFC technology to achieve information transmission and signal processing using external equipment. It can also treat the wound by releasing the antibacterial drug cefazolin on the wound surface by controlling the voltage of the drug delivery module. This wound care management technology can be used widely in the fields of multi-functional and personalized medicine and health care.

A flexible, breathable electronic device with real-time temperature sensing functions was proposed to monitor the infection or inflammation at the wound site and eliminate bacterial infection on demond by a thermally responsive fiber (Gong et al., 2019). The device was assembled from cross-linked electrospun moxifloxacin hydrochloride (MOX) loaded with a thermally responsive poly(N-isopropyl acrylamide-co-N-methylol acrylamide) (C-PNHM) nano-mesh film. The nano-mesh film contained a screen-printed conductive pattern (SC-PNHM) (Figure 4F). Lou et al. (2020) developed a flexible wound healing system (FWHS) to monitor the significant physiological process of wound healing and provide early warning and diagnosis of infection and wound invasion (Figure 4G). The system consisted of a double-layer: the upper layer included a flexible temperature sensor, a power-management circuit and a data processing circuit; the lower layer was composed of a collagen-chitosan dermal substitute. The system displayed good reliability and *in vitro* biocompatibility, as well as good accuracy, stability, and scalability. Zhang et al. (2021) proposed a flexible, integrated sensing platform (FISP) for monitoring local wound temperature as a reference for early prediction of pathological wound infection. The real-time wound temperature during each infected period was analyzed *via* multiple logarithmic regression. The higher the local temperature of the wound, the greater the risk of infection with gram-positive bacteria. In addition, the resulting data could be transmitted to the external device *via* Bluetooth. This work is expected to play an important role in wound diagnosis, remote treatment and artificial intelligence diagnosis.

### Other

Although there are researches for monitoring pathogens in wounds and treating infected wounds, the infection detection, and wound treatment are designed for broad-spectrum pathogen detection and inhibition (Lister et al., 2009; Bui et al., 2019; Edwards and Harding, 2004). Blind, untargeted administration will lead to additional side effects. To accurately and quantitatively obtain reliable POCT data such as *Pseudomonas aeruginosa* (*P. aeruginosa*), Brunauer et al. (2021) proposed a nucleic-acid lateral flow immunoassay approach to achieve rapid detection of specific infected wound pathogens. First, the gene DNA (gDNA) from the rough cleavage fluid was amplified by beading the pathogen. Then, the amplified product was detected using a nucleic acid lateral flow immunoassay. Rapid process chain analysis of pathogens could be completed *via* a simple diagnostic process. The time required for *P. aeruginosa* was less than 30 min and the lowest wound exudate detection limit was 2.1 × 10^5^ CFU/ml. Complex wounds are the result of slow healing due to a variety of environmental and physiological factors. These factors are reflected in the composition of the wound exudate, which includes a dynamic mixture of biochemical factors (cytokines, growth factors) and microorganisms during wound healing (Drinkwater et al., 2002). In order to better monitor potential multivariable pathological factors in chronic wounds and implement more personalized treatment strategies, Gao et al. (2021) proposed a flexible microfluidic multiple immunosensor platform for multivariate analysis of the wound microenvironment at care points (Figure 4H). The sensor system integrated a sensor array, a microflow wound exudate collector, and flexible electronic devices for real-time detection of inflammatory mediators (tumor necrosis factor-α, IL-6, IL-8 and transforming growth factor-β 1), the microbial load (*S. aureus*) and various physicochemical parameters (temperature and pH). The detection results can be read wirelessly. Thus, the data can be collected, analyzed, and visually referenced using external devices. This approach is expected to produce intelligent auxiliary dressings for clinical treatment and provide more personalized clinical diagnostic information.

As we all know, judging the condition of a wound based on single or partial biochemical information cannot replace standard pathological diagnosis. One cannot infer the wound infection stage and pathogen type using only a single reading of the wound pH, temperature, and pressure. However, the above-mentioned sensing technology designed for wound monitoring and healing can provide preliminary analysis of wound lesions during the window period before pathological diagnosis. This can reduce patient psychological burdens and mental stress. Although a variety of wearable sensor devices were designed for wound window diagnosis, research and development of multi-marker analysis sensor devices are required to provide more comprehensive real-time wound infection and healing information. In the future, an advanced generation of wearable devices will provide users or patients with more comprehensive and accurate real-time physiological information based on molecular or environmental signals and transmit the relevant information to a variety of applications for medical wound and health care management. Before these multi-functional and wearable sensors are used widely in clinics, they must pass a series of scientific and human application tests. There must also be a good understanding of the correlation between sensor information and a medical diagnosis. Therefore, substantial further research on intelligent, wearable sensor devices is required. Future studies may focus on material innovation and the development of a variety of analysis systems. We look forward to exciting new developments in this field shortly, as well as to continuous improvements in patient quality of life and the medical environment.

## Discussion, Conclusions, and Future Research

The purpose of this review is to summarize the opportunities provided by the development of intelligent, wearable sensors for healthcare and wound heal. We illustrate how researchers have designed intelligent, wearable sensors to collect and analyze target analytes from various biofluids. In addition to providing a tabulated summary of new biochemical sensing modalities and novel sensing platforms, we highlight the extension of the utility of these new monitoring platforms for assessing human health status and healthcare applications. These include achieving simultaneous monitoring of multiple informative metrics to detect specific diseases and expanding intelligent wearable sensors from the laboratory scale to a more natural clinical setting wherever possible. Since the advent of lab-scale intelligent sensing devices, health monitoring and wearable biochemical sensors have often been linked to the human skin and tissue interface. With the deeper implementation of related studies, other advanced sensing technologies, including microneedle sensing technology, will become an important part of future medical services. The microneedle sensing device can obtain and analyze biofluids painlessly and minimally invasively, which can avoid tissue damage and foreign body reactions to the greatest extent and is quite important for early human health monitoring and disease prevention. In addition, the future design of microneedle sensor needs to pay attention to the following points: After the device is implanted in the epidermis, in addition to monitoring the target analyte, it can also analyze and monitor the inflammatory response that may occur in the body due to the foreign body reaction, which may provide a reference for the development of minimally invasive microneedle devices with more precise and independent monitoring performance. Moreover, larger and more circumscribed improvements, such as the development of rapid, durable, reliable, and miniaturized sensor strategies, will be necessary for clinical analysis and the application of continuous health monitoring to chronic diseases and human health data. Meantime, the integrated analysis-diagnosis-treatment sensor device can provide great convenience for patients with self-care ability.

Although some progress has been made in developing advanced intelligent wearable sensors, there are still a series of challenges as follows: 1) Although most of the sensors with high sensitivity and high precision have good clinical application prospects, the performance of the sensor will degrade with the continuous operation, and the quality of the sensor still needs to be improved. At the same time, when the sensor continues to collect, transport, and analyze biological fluids, it is necessary to improve the reliability of the sensor and the consistency with the relevant target analyte concentration changes, to avoid frequent sensor replacement. 2) The accuracy and sensitivity of the sensor are also related to its surface fouling. If the sensor collects biological fluid with relatively complex components or turbid adhesion (such as pus exudated from infectious wounds), it may affect the regular operation of the sensor. Therefore, developing advanced sensing devices with surface antifouling properties and self-sensing calibration modes (multi-detection modes or multi-analyte sensors) is necessary. 3) Most intelligent wearable sensors can only detect target analytes in common biological fluids. People also need to develop the sensor systems for analysis of more other body fluids. For example, nipple secretion can be obtained directly in a non-invasive way, and the level of hormones and protein contained may strongly correlate with certain diseases. Nipple aspiratefluid steroid hormone levels and plasminogen activator inhibitors can be used as target analytes to detect breast cancer (Shidfar et al., 2016; Shaheed et al., 2017). 4) Some self-powered sensor devices can only meet the operational needs of the sensor itself. However, the sensor needs more energy supplement in data analysis, acquisition, and wireless communication. Therefore, it is urgent to integrate more efficient power supply methods. At present, energy storage devices (supercapacitors) (Wang et al., 2016), organic solar cells (O’Connor et al., 2016), biofuel cells (Jia et al., 2013), thermoelectric generators (Oh et al., 2016), and their composition are integrated to solve this challenge.

In the first half of this article, we chronicled recent advances in wearable intelligent sensing devices for personal healthcare and emphasized their advantages concerning achieving high precision, high sensitivity, and high stability health diagnoses. We gave an overview of various intelligent sensors (patches, dressings, microneedles, tattoos) and the related detection principles (colorimetry, security, probe assay, fluorescent signaling method). Judging from current areas of research interest, advances in microfluidic biosensors and electrochemically integrated sensors are focused on miniaturization design. This greatly enhances biosensor sensitivity, stability, and portability. Colorimetric biosensors are widely used for their visual readout features; wearable chemical and biosensors increasingly tend to energy autonomy. Cheap, simple, efficient wearable sensors can be made *via* inkjet, screen, and 3D printing technologies. Intelligent, wearable sensors can be used to monitor specially targeted analytes in raw fluids for early detection of human health changes. Blood remains the most authoritative biological fluid for human physical examination and screening. However, additional attention has been paid to more easily available, naturally secreted biofluids (sweat, interstitial fluid, tears, and wound fluid). Sample liquids are collected by an advanced sensor system and analyte information is collected. The results are transmitted to the user’s or patient’s interface either directly or *via* Bluetooth, NFC, or high-frequency passive RFID to provide appropriate information to patients, users, and doctors. However, the current challenge is that the associated communication often suffers from defects such as a low transmission rate or incompatible equipment. Therefore, other next-generation technologies, such as optical wireless technology, are needed urgently to develop information transmission algorithms and apply them to wearable devices.

The difficulty of nursing complex wounds should not be underestimated. Intelligent wound dressings are needed for diagnosis, treatment, monitoring, practical application, and sensor function. The second half of this paper summarized the application of emerging “smart + connected” wound sensing devices to monitor various target analytes from different types of wound models (i.e., infected, chronic, and acute wounds). It provided a new strategy for scientific wound care and reliable prediction. We believe that there will be more advanced and innovative scientific experiments and methods that can extend the concepts of wearable sensing devices to clinical medicine and health care. By reasonably weighing the public treatment strategies, this type of equipment can help a transition from a profit-based product model to a shared health care model based on the nature of public services. If this trend succeeds, it will be a vital achievement for the health care industry and will help users to have healthy lives.
